# Factorial calculation of calcium and phosphorus requirements of growing dogs

**DOI:** 10.1371/journal.pone.0220305

**Published:** 2019-08-02

**Authors:** Linda Franziska Böswald, Carmen Klein, Britta Dobenecker, Ellen Kienzle

**Affiliations:** Department of Veterinary Sciences, Chair of Animal Nutrition and Dietetics, Ludwig-Maximilians-Universität München, Munich, Germany; University of Bari, ITALY

## Abstract

Calcium and phosphorus requirements for growing dogs can be calculated by different methods. The current standard feeding recommendations are based on experimental data derived from young giant breed puppies. In order to determine the absolute requirement, an extrapolation via metabolisable energy requirement is recommended. Another approach is to calculate the requirement factorially, taking into account the endogenous losses and the amount of calcium and phosphorus retained due to tissue accretion during growth as well as the expected availability of these nutrients. The working hypothesis was that both methods are valid and lead to comparable results in young puppies of a high mature body weight (BW). Yet, deviations for other age and mature BW groups were expected. Thus, the aim of the present study was to compare the results of both methods using exemplary puppies of different age and mature BW groups. The hypotheses could be verified for calcium. The extrapolated requirements overestimate the factorial requirements by up to 59.7% for puppies <60kg mature BW and/or >6 months of age. In case of phosphorus requirement, the deviations between both methods are overall very high in all stages. Taking into account the potentially harmful effects of calcium and phosphorus excess, the feeding recommendations based on the extrapolation should be reconsidered.

## Introduction

For growing dogs, the supply with calcium and phosphorus is essential for a healthy development. In dogs, intestinal calcium absorption cannot be adapted to supply [1–4]. Deficiency [5,6] as well as excess [7–10] can lead to disorders in skeletal development (review by [11]). Phosphorus deficiency during growth has been shown to lead growth reduction and disturbance in musculoskeletal development [12,13]. Especially large and giant breed puppies are at risk of developing skeletal disorders during growth [14–17].

In order to prevent skeletal disorders related to calcium and phosphorus supply, reliable feeding recommendations are important. In the species dog, it is complicated to formulate “general” recommendations because there are vast differences between breeds regarding mature size and rate of weight gain [18–20].

Nutrient requirements are not fixed values but estimations which include safety margins of different dimensions. They should not be applied without critical assessment and should be adapted when new research is available [21]. There are different ways to establish nutrient recommendations and it cannot generally be said that one approach or model is generally correct. In case of a species or nutrient with little information available, trial and error might be the first approach, resulting in a roughly defined area of acceptable nutrient supply. Dose-response relationship experiments are more elaborate and help to narrow down the level of nutrient supply that is adequate in the given conditions. With graded doses of the nutrient in question, the effect on the animals is documented and the optimum is defined as the medium dose without negative effects of either deficiency or intoxication (e.g. review by [22]). This approach is limited because the effect of a certain level of nutrient supply is often linked to other conditions (animal age, performance, environment as well as feed composition and processing).

The mineral requirements for growing puppies after weaning given by the widely accepted NRC [23] have been established by extrapolating from feeding trials with different dietary nutrient content, which is more or less a trial and error approach. These values have been derived from experimental data which, in the case of calcium, stems mostly from research with large breed puppies. Great Danes were used by Hazewinkel et al. [16], Goodman et al. [24], Lauten & Goodman [25] and Schoenmakers et al. [11], while Laflamme [26] used medium- to large-breed puppies and only Nap et al. [27] give data on a smaller breed, i.e. miniature poodles. For phosphorus requirements, it is similar: German Shepherds [12] and Great Danes [11] were used for extrapolation to other breeds. The possible overestimation of the need of small- or medium-sized breeds is commented on [23].

In the NRC [23], the recommended daily allowance (RDA) for calcium and phosphorus for growing dogs are given in the unit g/kg body weight (BW)^0.75^ for one exemplary situation of a 5.5kg puppy with an expected mature BW of 35kg. For all other situations, the unit g/1000kcals of metabolisable energy (ME) requirement, which can also be calculated according to the NRC, is specified.

Another method for establishing nutrient requirements is the factorial calculation (e.g. [21,28–30]). Endogenous losses via skin, urine and faeces have to be quantified and summed up [31]. Taking into account the expected availability of the nutrient in question, the minimum requirement for maintenance to compensate for the endogenous losses can be estimated. For any kind of performance (e.g. growth, gestation, lactation), the extra amount of nutrient loss or gain is calculated and added to the maintenance net requirement. In case of growth, this would be the amount of the nutrient in the body tissue gain. The net requirement divided by the expected availability renders the “gross” requirement, the amount of a nutrient that has to be fed. In this calculation, the estimation of availability is critical because it is the denominator of the fraction and small changes of estimated availability can alter the feeding recommendations vastly.

The benefit of the factorial calculation of calcium and phosphorus requirements of growing dogs is that differences in growth rates between small and large dogs can be taken into account.

The aim of the present study was to compare the extrapolation [23] and the factorial calculation of calcium and phosphorus requirements for growing dogs. The first hypothesis was that both methods should result in comparable values for young giant breed puppies because NRC data was derived mostly from animals of this group. The second hypothesis was that for other age and mature BW groups, there would be deviations.

To test the hypotheses, calcium and phosphorus requirements for exemplary puppies of different age and mature BW groups were calculated according to NRC [23] and the factorial method and compared.

## Material and methods

Ethical approval by the commission of the Veterinary Faculty of the LMU München was obtained (144-28-08-2018).

### Body weight development

Expected BWs (kg) and the respective metabolic BW (kg^0.75^) for the ages 9, 13, 17, 22, 26, 31, 35, 39, 44, 48 and 52 weeks were calculated for exemplary puppies with mature BWs of 5, 10, 20, 35 and 60kg according to literature data [32, 33], based on [31] and [34, 35] (see S1 Table). Data given for the end of a month was transferred to weeks as follows: weeks = months ∙ 30.5 / 7. For the ages in between the given age groups (5–6 and 7–12 months), an extrapolation was conducted. If the mature BW was reached before the age of 52 weeks, this indicated a finished growth period and no requirements for growth were calculated.

### Calculation of requirements according to NRC

The absolute calcium and phosphorus requirement for growing dogs after weaning in the NRC [23] needs to be extrapolated via ME requirement for all puppies differing from the example situation that can be calculated with g per kg BW^0.75^ (current BW 5.5kg, mature BW 35kg). The ME requirement for each age and mature BW stage was calculated according to the equation by the NRC [23]: ME requirement (kcal) = 130 · kg BW^0.75^ · 3.2 · (e^(-0.87p)^– 0.1) with p = current BW / mature BW and e = 2.718.

Then, the recommended calcium and phosphorus supply was calculated with the recommended daily allowance (RDA) for puppies after weaning of 3g and 2.5g / 1000kcal of ME requirement, respectively.

### Factorial calculation of requirements

The factorial requirements for growth were calculated using data on average daily gain (ADG) and presumed availability from the German Society of Nutrition Physiology [31] adapted according to Dobenecker 2002 [1]. Body composition of puppies according to Kienzle et al. [36] was used to calculate the total body content of calcium and phosphorus for each age and mature BW group and the content in the gained tissue. Faecal endogenous losses were calculated from literature data [37–43] and [Dobenecker unpublished] on growing dogs´ calcium and phosphorus intake and faecal excretion via modified Lucas tests (previously described by [2] and [3] for adult animals). This resulted in the estimation of endogenous calcium losses of 30mg/kg BW and endogenous phosphorus losses of 26mg/kg BW in growing dogs.

For each age and mature BW group, the factorial requirement of calcium and phosphorus was calculated with the following equation: requirement (mg / day) = (ADG · nutrient content in ADF + E) · 100 / availability (%) with E = faecal endogenous losses.

## Results

The S2 and S3 Tables give the absolute calcium and phosphorus requirement in mg per day for the exemplary age and mature BW groups calculated according to the factorial approach and the RDA by NRC [23]. For the 52 weeks old dog with a mature BW of 5kg, no value was calculated because the mature BW is reached and a requirement for growth would be inaccurate.

**Fig 1:**
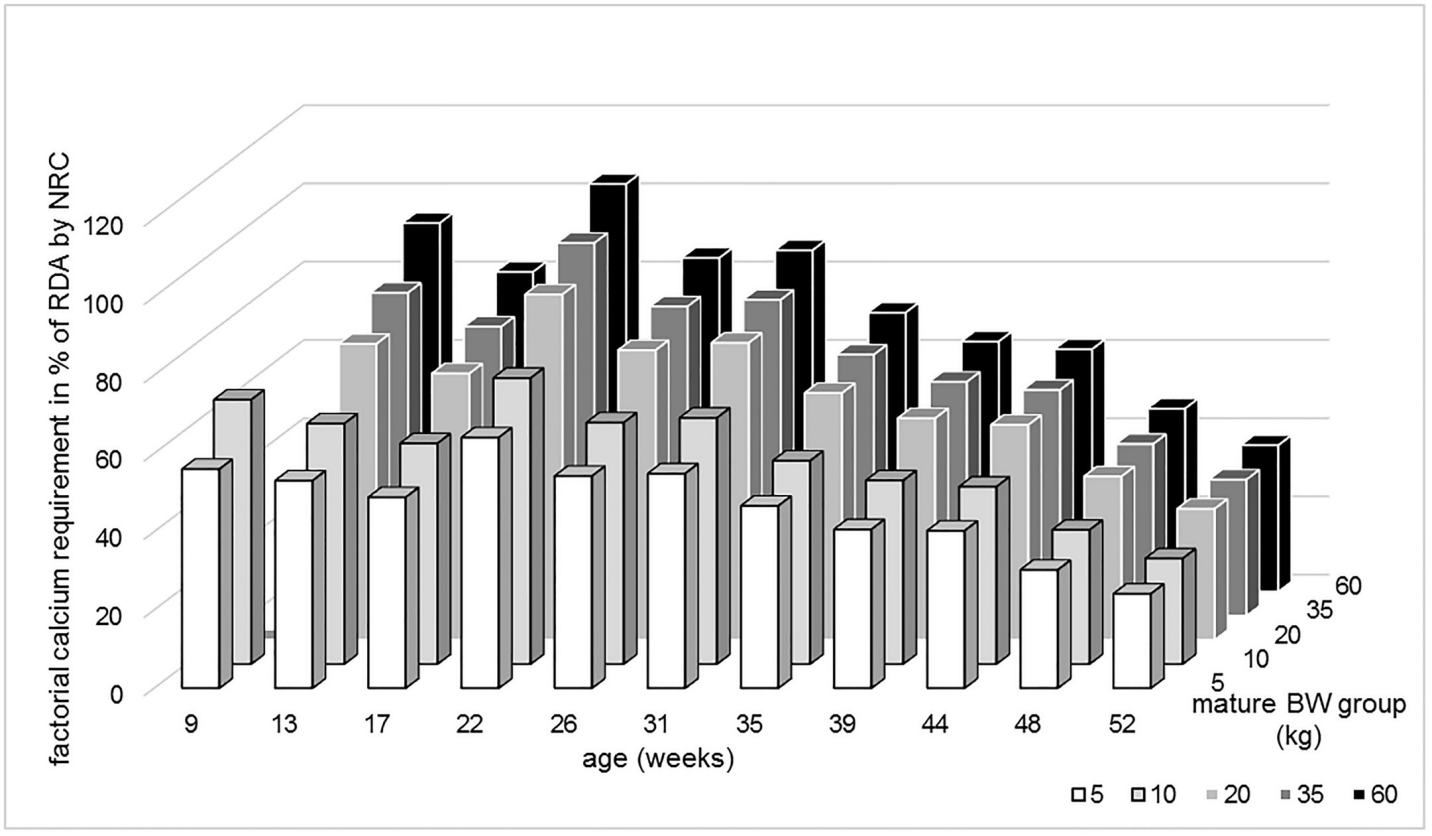
Comparison of calcium requirement calculated according to NRC and the factorial approach. The columns describe the factorially calculated requirement as percentage of the recommended daily allowance by NRC [23].

The comparison of calcium requirements (see Fig 1 and S4 Table) shows that the results of both methods are similar for the high mature BW groups under the age of 6 months (up to 94.2% agreement in the 13 weeks old puppies of 60kg mature BW). The deviation increases with lower mature BW and increasing age.

**Fig 2:**
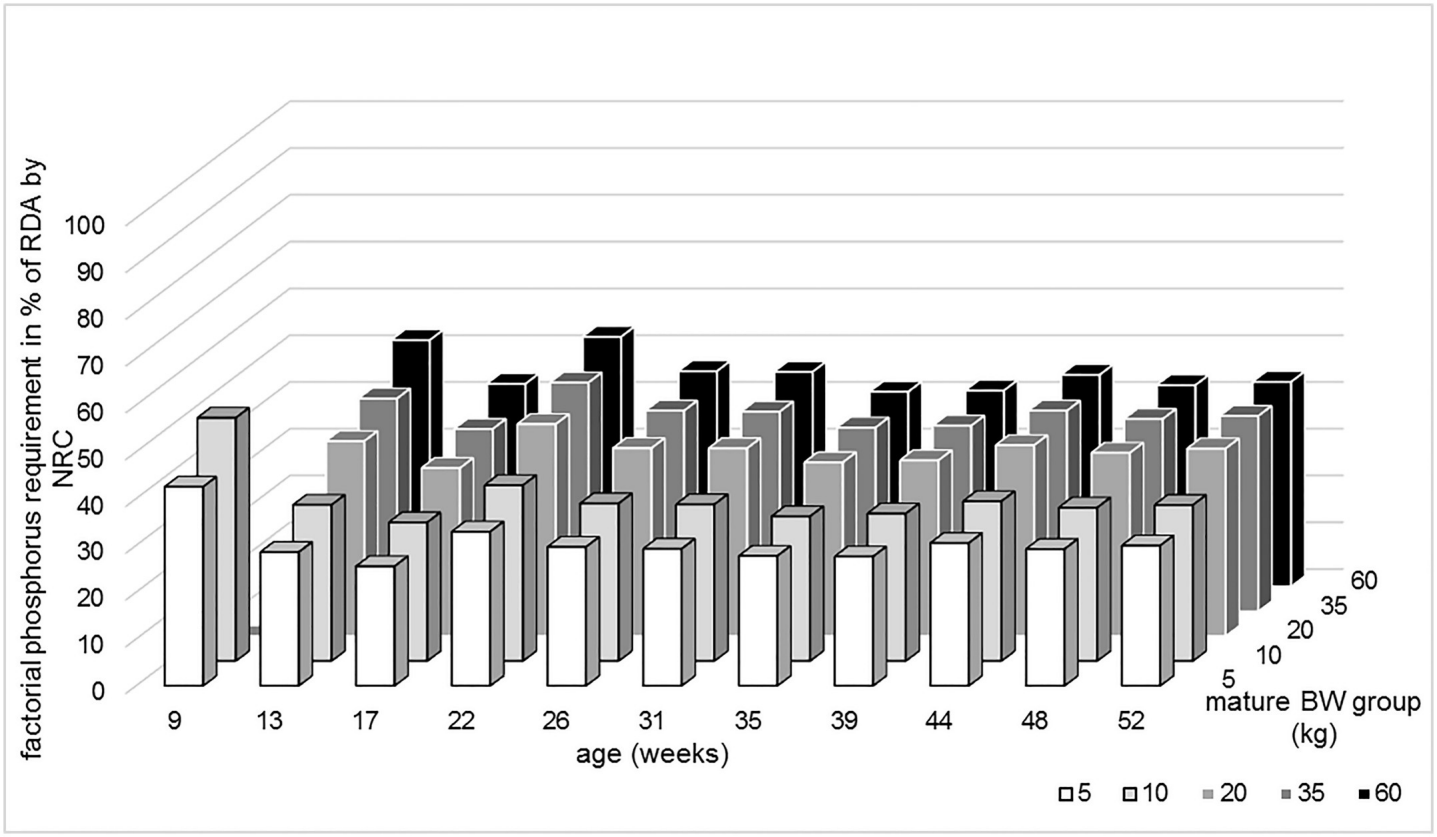
Comparison of phosphorus requirement calculated according to NRC and the factorial approach. The columns describe the factorially calculated requirement as percentage of the recommended daily allowance (RDA) by NRC [23].

For phosphorus, the deviation of factorially calculated phosphorus requirement and the NRC [23] requirement (Fig 2, S4 Table) is rather high for all age and mature BW groups with 52.7% being the best agreement in the 13 weeks old puppies with a mature BW of 60kg.

## Discussion

Calcium requirements for giant breed puppies (mature BW 60kg) under the age of 7 months were in good agreement between the extrapolation [23] and the factorial approach. Data of Great Danes in this age group is the basis of the NRC [23] extrapolation [11,15,24,25]. Thus, the match of both methods for this mature BW and age group suggests that both methods are valid.

In miniature and medium size puppies (5-35kg mature BW), the extrapolated NRC [23] recommendations are markedly higher than the factorially derived calcium requirements (see Fig 1). Large breed puppies have a higher growth intensity than smaller ones, i.e. they gain more weight per kg BW^0.75^ over the same time period. The extrapolation from giant breed to medium and small breed puppies overestimates the nutrient requirement of the smaller dogs.

Calcium excess during growth seems to have detrimental effects on skeletal health, especially in combination with fast growth [7,8] (reviewed by [10]). Even in beagle puppies raised with restricted energy supply, a subclinical effect on the long bones, i.e. premature closure of the growth plates, has been observed [9]. New data on calcium homeostasis in adult dogs shows that high calcium supply might lead to a downregulation of bone turnover [3]. However, the exact mechanism is not completely understood. The risk of skeletal disease is especially high when calcium excess, i.e. slow bone turnover, meets too fast growth due to energy oversupply [10,17]. The NRC [23] offers a safe upper limit (SUL) of calcium intake in growing dogs which is 1.5 times the RDA (4.5g / 1000kcal ME). If this SUL is applied by multiplying the factorially calculated calcium requirement by 1.5, the NRC recommendations for calcium intake are above this range in puppies with a mature BW of 5kg or older than 35 weeks. The same is true for puppies with a mature BW of 10kg aged ≥13 weeks and puppies with a mature BW of 20kg aged ≥35 weeks.

For phosphorus, the deviation between NRC [23] and factorial requirements is even larger. In the NRC recommendation, the RDA for phosphorus is set for a calcium/phosphorus ratio of 1.2/1 without variation in either variable. In the factorial calculation, the requirement for both nutrients is calculated independently with regard to the concentrations in the gain of tissue. The calcium/phosphorus ratio of the gained tissue is ~2/1 during the period of maximum growth (2–4 months) and decreases to 1.49 in 7–12 months old dogs [31,36]. The higher ratio in the younger puppies may indicate the period of skeletal development because bone has a calcium/phosphorus ratio of ~1.8/1-2/1 [44] while muscle and fat gain during the later growth leads to a higher relative phosphorus concentration in the gained tissue.

It is important to note that the factorially calculated requirements lead to a variation in the resulting calcium/phosphorus ratio during growth. The maximum calcium/phosphorus ratio calculated from the factorially calculated requirements in the given age groups was 2/1–2.3/1 in the age groups 13–35 weeks. This corresponds with the period of highest growth intensity. Afterwards, the calcium/phosphorus ratio decreases because during this growth period there is more gain of non-skeletal body mass, which contains relatively more phosphorus than calcium. The variation in calcium/phosphorus ratios derived from the factorial calculation can be explained with these physiological processes, but whether it should be recommended for practical feeding, remains unclear. It is not known and cannot be predicted what the actual effects of a variation of calcium/phosphorus ratio in contrast to a constant ratio will be. There might be metabolic effects of higher or lower calcium/phosphorus ratios that cannot be gauged by factorial calculation of requirements. In growing pigs, poultry and cattle, the calcium/(digestible) phosphorus ratio is kept rather constant throughout the rearing and fattening period [45,46,47]. To be on the safe side, it might be better to recommend an ideal calcium/phosphorus ratio of 1.4/1 throughout growth for dogs.

As expected, there were deviations between the factorially calculated calcium and phosphorus requirements and the extrapolated recommendations by the NRC [23]. The NRC [23] recommendations for calcium overestimated the factorial calculation significantly for puppies with a mature BW of ≤ 20kg and/or older than 7 months. The factorial phosphorus requirement was vastly overestimated in all age and mature BW groups.

The factorial requirements allow for a gradual decrease of calcium and phosphorus requirements after the main growth phase and a smooth transition to adult maintenance requirements. As an example, the maintenance calcium requirement of an adult dog of 20kg BW stated by the NRC is 1229mg/d (0.13g/kg BW^0.75^). The calcium requirement of a 52-weeks-old puppy with a mature BW of 20kg is 2113mg/d according to the factorial calculation and 3810mg/d according to the NRC [23] extrapolation. The factorial requirement is clearly closer in the range of the adult requirement than the extrapolated, higher recommendation.

For farm animals which have different requirements for their respective performance (e.g. gestation, lactation, egg production, fattening), the factorial method is well established to calculate the individual energy and nutrient requirements [21,26,44].

The factorial calculation of nutrient requirements should be performed to serve as a verification of the extrapolation from experimental data. It is recommended to compare the results of both methods. The ideal situation is when both calculations give matching results, as seen in the calcium requirement of giant breed puppies (Fig 1). Extrapolated results that are below the factorially derived net requirement would not be plausible and must be flawed. If the factorial gross requirement is much higher than the experimental data suggests, a below-average availability may have been assumed in the factorial calculation. In any case, a high deviation between the results of both calculations must lead to an evaluation of plausibility of the recommendations.

Given the current state of knowledge, the factorially calculated requirements for calcium and phosphorus should be used for dogs. The required data on availability, BW development and composition of gained tissue is available for growing dogs, while the NRC [23] extrapolation lacks data on medium and small breed puppies. It is known that an oversupply can have negative effects on the growing dogs´ health. Because of possible metabolic effects of a variation of calcium/phosphorus ratios, a constant ratio of ca. 1.4/1 should be aimed at.

## Supporting information

S1 Table Body weight. Expected body weight (kg) of exemplary puppies of different mature body weight groups at different ages (calculated according to Klein et al. [35]).
(DOCX)

S2 Table Factorial requirement. Absolute calcium and phosphorus requirement (mg/d) for puppies of different age and mature body weight groups calculated according to the factorial approach.
(DOCX)

S3 Table Extrapolated requirement. Absolute calcium and phosphorus requirement (mg/d) of growing dogs of different age and mature body weight groups, calculated according to the recommended allowance per 1000kcal metabolisable energy requirement by NRC [23].
(DOCX)

S4 Table Comparison. Factorial calcium and phosphorus requirement expressed as % of NRC [23] recommendation for growing dogs.
(DOCX)

S5 Table Relative factorial requirement. Calcium and phosphorus requirement (mg/kg BW0.75) for puppies of different age and mature body weight groups calculated according to the factorial approach.
(DOCX)
