# Supplementary material for: Factorial calculation of calcium and phosphorus requirements of growing dogs
Source: PLoS One. 2019 Aug 2;14(8):e0220305. doi: 10.1371/journal.pone.0220305 (PMC6677383; doi:10.1371/journal.pone.0220305)
Supplement: S1 Table — Expected body weight (kg) of exemplary puppies of different mature body weight groups at different ages (calculated according to Klein et al. [35]). (DOCX) [file pone.0220305.s001.docx]

**S1 Table.** **Body weight.** Expected body weight (kg) of exemplary puppies of different mature body weight groups at different ages (calculated according to Klein et al. [35]).

| **mature body weight** *(kg)* | **5** | **10** | **20** | **35** | **60** |
| --- | --- | --- | --- | --- | --- |
| **age** *(weeks)* |  |  | *kg* |  |  |
| **9** | 1.88 | 3.28 | 5.38 | 8.76 | 10.83 |
| **13** | 2.56 | 4.63 | 8.32 | 13.51 | 18.91 |
| **17** | 3.05 | 5.62 | 10.46 | 16.98 | 24.80 |
| **22** | 3.53 | 6.57 | 12.51 | 20.32 | 30.46 |
| **26** | 3.84 | 7.19 | 13.85 | 22.48 | 34.13 |
| **31** | 4.16 | 7.84 | 15.25 | 24.75 | 38.00 |
| **35** | 4.39 | 8.28 | 16.22 | 26.32 | 40.66 |
| **39** | 4.58 | 8.68 | 17.08 | 27.72 | 43.04 |
| **44** | 4.81 | 9.13 | 18.04 | 29.28 | 45.69 |
| **48** | 4.97 | 9.45 | 18.73 | 30.41 | 47.60 |
| **52** | – | 9.74 | 19.37 | 31.44 | 49.36 |
