# Supplementary material for: Factorial calculation of calcium and phosphorus requirements of growing dogs
Source: PLoS One. 2019 Aug 2;14(8):e0220305. doi: 10.1371/journal.pone.0220305 (PMC6677383; doi:10.1371/journal.pone.0220305)
Supplement: S2 Table — Absolute calcium and phosphorus requirement (mg/d) for puppies of different age and mature body weight groups calculated according to the factorial approach. (DOCX) [file pone.0220305.s002.docx]

**S2 Table. Factorial requirement.** Absolute calcium and phosphorus requirement (mg/d) for puppies of different age and mature body weight groups calculated according to the factorial approach.

| **mature body weight** *(kg)* | **5** | **10** | **20** | **35** | **60** |
| --- | --- | --- | --- | --- | --- |
| **age** *(weeks)* | *calcium mg /d* | | | | |
| **9** | 697 | 1339 | – | – | – |
| **13** | 724 | 1376 | 2757 | 4464 | 7040 |
| **17** | 686 | 1319 | 2642 | 4286 | 6777 |
| **22** | 727 | 1392 | 2817 | 4583 | 7323 |
| **26** | 766 | 1471 | 2970 | 4806 | 7660 |
| **31** | 768 | 1492 | 3036 | 4925 | 7939 |
| **35** | 645 | 1223 | 2506 | 4063 | 6514 |
| **39** | 556 | 1096 | 2231 | 3626 | 5842 |
| **44** | 581 | 1117 | 2280 | 3703 | 6002 |
| **48** | 538 | 1039 | 2117 | 3448 | 5543 |
| **52** | – | 1030 | 2114 | 3422 | 5520 |
| **age** *(weeks)* | *phosphorus mg /d* | | | | |
| **9** | 441 | 859 | – | – | – |
| **13** | 325 | 623 | 1266 | 2049 | 3278 |
| **17** | 299 | 557 | 1161 | 1884 | 2994 |
| **22** | 324 | 621 | 1255 | 2041 | 3256 |
| **26** | 349 | 669 | 1342 | 2173 | 3444 |
| **31** | 341 | 661 | 1338 | 2170 | 3478 |
| **35** | 320 | 606 | 1229 | 1993 | 3168 |
| **39** | 315 | 613 | 1234 | 2004 | 3190 |
| **44** | 343 | 656 | 1324 | 2149 | 3436 |
| **48** | 325 | 624 | 1258 | 2046 | 3256 |
| **52** | – | 628 | 1273 | 2063 | 3294 |
