# Supplementary material for: Factorial calculation of calcium and phosphorus requirements of growing dogs
Source: PLoS One. 2019 Aug 2;14(8):e0220305. doi: 10.1371/journal.pone.0220305 (PMC6677383; doi:10.1371/journal.pone.0220305)
Supplement: S3 Table — Absolute calcium and phosphorus requirement (mg/d) of growing dogs of different age and mature body weight groups, calculated according to the recommended allowance per 1000kcal metabolisable energy requirement by NRC [23]. (DOCX) [file pone.0220305.s003.docx]

**S3 Table. Extrapolated requirement.** Absolute calcium and phosphorus requirement (mg/d) of growing dogs of different age and mature body weight groups, calculated according to the recommended allowance per 1000kcal metabolisable energy requirement by NRC [23].

| **mature body weight** *(kg)* | **5** | **10** | **20** | **35** | **60** |
| --- | --- | --- | --- | --- | --- |
| **age** *(weeks)* |  |  | *calcium mg /d* |  |  |
| **9** | 1244 | 1982 | 3048 | 4476 | 5623 |
| **13** | 1365 | 2239 | 3646 | 5407 | 7472 |
| **17** | 1406 | 2338 | 3880 | 5801 | 8294 |
| **22** | 1418 | 2380 | 3988 | 6014 | 8786 |
| **26** | 1413 | 2384 | 4009 | 6081 | 8982 |
| **31** | 1399 | 2372 | 3998 | 6101 | 9100 |
| **35** | 1385 | 2355 | 3973 | 6089 | 9135 |
| **39** | 1370 | 2335 | 3940 | 6062 | 9139 |
| **44** | 1350 | 2307 | 3892 | 6016 | 9115 |
| **48** | 1334 | 2284 | 3852 | 5974 | 9081 |
| **52** | – | 2261 | 3810 | 5928 | 9038 |
| **age** *(weeks)* | *phosphorus mg /d* | | | | |
| **9** | 1037 | 1652 | 2540 | 3730 | 4686 |
| **13** | 1138 | 1866 | 3038 | 4505 | 6226 |
| **17** | 1172 | 1949 | 3233 | 4834 | 6911 |
| **22** | 1181 | 1983 | 3323 | 5011 | 7322 |
| **26** | 1177 | 1986 | 3341 | 5067 | 7485 |
| **31** | 1166 | 1976 | 3332 | 5084 | 7583 |
| **35** | 1154 | 1963 | 3311 | 5074 | 7613 |
| **39** | 1142 | 196 | 3283 | 5052 | 7616 |
| **44** | 1125 | 1822 | 3243 | 5014 | 7596 |
| **48** | 1112 | 1903 | 3210 | 4978 | 7567 |
| **52** | – | 1884 | 3175 | 4940 | 7531 |
