# Supplementary material for: Factorial calculation of calcium and phosphorus requirements of growing dogs
Source: PLoS One. 2019 Aug 2;14(8):e0220305. doi: 10.1371/journal.pone.0220305 (PMC6677383; doi:10.1371/journal.pone.0220305)
Supplement: S4 Table — Factorial calcium and phosphorus requirement expressed as % of NRC [23] recommendation for growing dogs. (DOCX) [file pone.0220305.s004.docx]

**S4 Table.** **Comparison.** Factorial calcium and phosphorus requirement expressed as % of NRC [23] recommendation for growing dogs.

| **mature body weight** *(kg)* | **5** | **10** | **20** | **35** | **60** |
| --- | --- | --- | --- | --- | --- |
| **age** *(weeks)* | *calcium* | | | | |
| **9** | 56.0 | 67.6 | – | – | – |
| **13** | 53.0 | 61.5 | 75.6 | 82.6 | 94.2 |
| **17** | 48.8 | 56.4 | 68.1 | 73.9 | 91.7 |
| **22** | 51.3 | 58.5 | 70.7 | 76.2 | 93.3 |
| **26** | 54.2 | 61.7 | 74.1 | 79.0 | 85.3 |
| **31** | 54.9 | 62.9 | 75.9 | 80.7 | 87.2 |
| **35** | 46.6 | 51.9 | 63.1 | 66.7 | 71.3 |
| **39** | 40.6 | 46.9 | 56.6 | 59.8 | 63.9 |
| **44** | 43.0 | 48.4 | 58.6 | 61.5 | 95.8 |
| **48** | 40.3 | 45.5 | 55.0 | 57.7 | 61.0 |
| **52** | – | 45.6 | 55.5 | 57.7 | 61.1 |
| **age** *(weeks)* | *phosphorus* | | | | |
| **9** | 42.5 | 52.0 | – | – | – |
| **13** | 28.6 | 33.4 | 41.7 | 45.5 | 52.7 |
| **17** | 25.5 | 29.6 | 35.9 | 39.0 | 43.3 |
| **22** | 27.4 | 31.3 | 37.8 | 40.7 | 44.5 |
| **26** | 29.7 | 33.7 | 40.2 | 42.9 | 46.0 |
| **31** | 29.3 | 33.5 | 40.1 | 42.7 | 45.9 |
| **35** | 27.8 | 30.9 | 37.1 | 39.3 | 41.6 |
| **39** | 27.6 | 31.5 | 37.6 | 39.7 | 41.9 |
| **44** | 30.5 | 34.1 | 40.8 | 42.9 | 45.2 |
| **48** | 29.2 | 32.8 | 39.2 | 41.1 | 43.0 |
| **52** | – | 33.3 | 40.1 | 41.8 | 43.7 |
