# Supplementary material for: Factorial calculation of calcium and phosphorus requirements of growing dogs
Source: PLoS One. 2019 Aug 2;14(8):e0220305. doi: 10.1371/journal.pone.0220305 (PMC6677383; doi:10.1371/journal.pone.0220305)
Supplement: S5 Table — Calcium and phosphorus requirement (mg/kg BW0.75) for puppies of different age and mature body weight groups calculated according to the factorial approach. (DOCX) [file pone.0220305.s005.docx]

**S5** **Table. Relative factorial requirement.** Calcium and phosphorus requirement (mg/kg BW^0.75^) for puppies of different age and mature body weight groups calculated according to the factorial approach.

| **mature body weight** *(kg)* | **5** | **10** | **20** | **35** | **60** |
| --- | --- | --- | --- | --- | --- |
| **age** *(weeks)* |  |  | *calcium mg /d* |  |  |
| **9** | 434 | 550 | – | – | – |
| **13** | 358 | 436 | 563 | 634 | 776 |
| **17** | 297 | 361 | 454 | 512 | 610 |
| **22** | 282 | 339 | 525 | 479 | 565 |
| **26** | 279 | 335 | 515 | 466 | 542 |
| **31** | 263 | 316 | 393 | 444 | 519 |
| **35** | 213 | 251 | 310 | 350 | 405 |
| **39** | 178 | 217 | 266 | 300 | 348 |
| **44** | 179 | 213 | 261 | 294 | 342 |
| **48** | 162 | 193 | 235 | 266 | 306 |
| **52** | – | 187 | 229 | 258 | 296 |
| **age** *(weeks)* | *phosphorus mg /d* | | | | |
| **9** | 275 | 352 | – | – | – |
| **13** | 161 | 197 | 258 | 291 | 362 |
| **17** | 130 | 158 | 200 | 225 | 269 |
| **22** | 126 | 151 | 189 | 213 | 251 |
| **26** | 127 | 152 | 187 | 210 | 244 |
| **31** | 117 | 141 | 173 | 196 | 227 |
| **35** | 106 | 124 | 152 | 172 | 197 |
| **39** | 101 | 121 | 147 | 166 | 190 |
| **44** | 106 | 125 | 151 | 171 | 196 |
| **48** | 98 | 116 | 140 | 158 | 180 |
| **52** | – | 114 | 138 | 155 | 177 |
